# Finding the First Potential Inhibitors of Shikimate Kinase from Methicillin Resistant *Staphylococcus aureus* through Computer-Assisted Drug Design

**DOI:** 10.3390/molecules26216736

**Published:** 2021-11-08

**Authors:** Lluvia Rios-Soto, Alfredo Téllez-Valencia, Erick Sierra-Campos, Mónica Valdez-Solana, Jorge Cisneros-Martínez, Marcelo Gómez Palacio-Gastélum, Adriana Castillo-Villanueva, Claudia Avitia-Domínguez

**Affiliations:** 1 Facultad de Medicina y Nutrición, Universidad Juárez del Estado de Durango, Av. Universidad y Fanny Anitua S/N, Durango 34000, Mexico; lluviarios.soto@gmail.com (L.R.-S.); jorgecisner10@yahoo.mx (J.C.-M.); 2 Facultad de Ciencias Químicas, Universidad Juárez del Estado de Durango, Av. Artículo 123 S/N Fracc. Filadelfia, Gómez Palacio, Durango 35010, Mexico; ericksier@gmail.com (E.S.-C.); valdezandyval@gmail.com (M.V.-S.); 3 Facultad de Odontología, Universidad Juárez del Estado de Durango, Predio Canoas S/N, Los Angeles, Durango 34070, Mexico; gporthodgo@yahoo.com.mx; 4 Laboratorio de Bioquímica-Genética, Instituto Nacional de Pediatría, Secretaría de Salud, Ciudad de Mexico 04530, Mexico; acastilloinp@gmail.com

**Keywords:** MRSA, shikimate kinase, virtual screening, molecular dynamics, ADME-Tox properties

## Abstract

Methicillin-resistant *Staphylococcus aureus* (MRSA) is an important threat as it causes serious hospital and community acquired infections with deathly outcomes oftentimes, therefore, development of new treatments against this bacterium is a priority. Shikimate kinase, an enzyme in the shikimate pathway, is considered a good target for developing antimicrobial drugs; this is given because of its pathway, which is essential in bacteria whereas it is absent in mammals. In this work, a computer-assisted drug design strategy was used to report the first potentials inhibitors for Shikimate kinase from methicillin-resistant *Staphylococcus aureus* (SaSK), employing approximately 5 million compounds from ZINC15 database. Diverse filtering criteria, related to druglike characteristics and virtual docking screening in the shikimate binding site, were performed to select structurally diverse potential inhibitors from SaSK. Molecular dynamics simulations were performed to elucidate the dynamic behavior of each SaSK–ligand complex. The potential inhibitors formed important interactions with residues that are crucial for enzyme catalysis, such as Asp37, Arg61, Gly82, and Arg138. Therefore, the compounds reported provide valuable information and can be seen as the first step toward developing SaSK inhibitors in the search of new drugs against MRSA.

## 1. Introduction

Today, antimicrobial resistance in bacteria has become a serious healthcare concern [1]; the World Health Organization (WHO) published a list of 12 bacteria in 2017 [2], their level of resistance to antibiotics has become so grave, that they represent an important threat. Therefore, these organisms have been declared priority pathogens to encourage antibiotic drug design by pharmaceutical companies [3]. From the Gram-positive bacteria shown on this list, drug-resistant *Staphylococcus aureus* has become one of the most problematic pathogens worldwide.

Methicillin-resistant *Staphylococcus aureus* (MRSA) has been widely known as a major cause of nosocomial and community-acquired infections that range from mild cases, such as skin and soft tissue infections, to more serious and deadlier, like bacteremia, osteomyelitis, and infective endocarditis [4]. In 2017, an estimated of 12,000 cases of MRSA infections and 20,000 deaths associated with it, occurred in the United States alone [5]. Over the years, MRSA has shown an alarming increase in antimicrobial resistance [6,7,8], developing resistance to vancomycin as well, the last resort treatment for MRSA infections [9]. Furthermore, the recent underdevelopment trend of new antimicrobial agents by pharmaceutical companies has added to the threat that this pathogen already presents [10,11]. Moreover, it has become apparent that research and discovery of new drugs, that possess unique mechanisms of action to prevent chances of developing drug resistance, are becoming urgently necessary.

Accordingly, pathways that are essential for bacterial survival, absent in humans, have become ideal targets to obtain new antimicrobial agents. The shikimate pathway (SP) enzymes are attractive targets for the development of antimicrobial drugs, given that this pathway is only present in bacteria, plants, fungi, apicomplexan parasites, and it is absent in mammals [12]. SP links carbohydrate metabolism to aromatic compounds biosynthesis by means of seven metabolic steps culminating in chorismate production, an important precursor for the synthesis of aromatic amino acids, folate, ubiquinone, among other essential molecules [13]. This route has been previously validated as an antimicrobial target [14], therefore, the design of inhibitors against enzymes of this route has gained significant attention over the years [15].

Shikimate kinase (SK, EC 2.7.1.71) is the fifth enzyme in SP, it catalyzes the conversion of shikimate to shikimate 3-phosphate by phosphorylation of the 3-hydroxyl group of shikimates using ATP as co-substrate; this enzyme has already been established as a promising target, it is essential in a variety of organisms like *Mycobacterium tuberculosis*, where the deletion of the *aro*K gene, which codes for SK, disrupts cell viability [16]. SK has been recognized as a member of the nucleoside monophosphate kinases (NMP) family; it possesses three domains, the CORE domain, which involves residues that belong to the conserved binding loop (P-loop) which forms the binding site of ATP and ADP, the LID domain that closes over the active site and contains important residues for ATP binding, and the NMP-binding domain, an important region that corresponds to the shikimate binding region [17]. In 2019, our group reported the biochemical, kinetic, and structural characterization of SK from MRSA (SaSK). In that work, it was found that SaSK shares common characteristics with other bacterial SKs, and by performing homology modeling and molecular dynamics studies important structural information was reported [18].

This study was initiated to work with a chemical library of around 5 million small molecules under a computer-assisted drug design strategy; it included virtual screening, ADME-Tox properties predictions, and molecular dynamics simulations; it was completed to report the first set of potential inhibitors from SaSK.

## 2. Results and Discussion

### 2.1. Compounds Filtering

**Figure 1:**
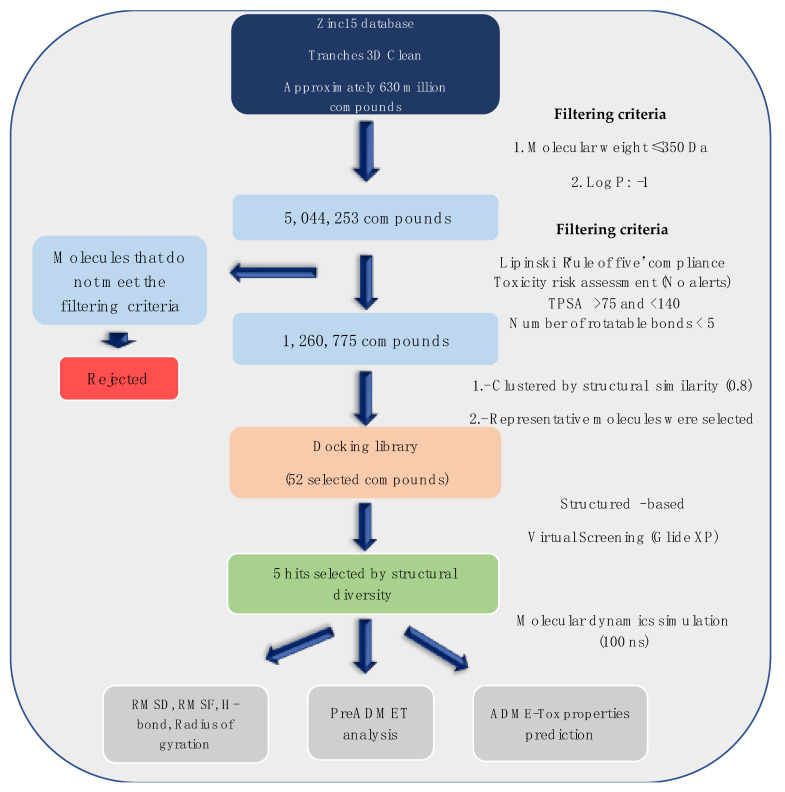
Workflow to select the potential SaSK inhibitors by computer-assisted drug design.

It is increasingly becoming apparent that toxic properties are crucial determinants for the successful development of new drugs. There are unfavorable characteristics that may lead to rejection of possible candidates in the later stages of the drug process [19]. In this study, a total of 5,044,253 compounds, based on SaSK substrate molecular weight and Log *p* value, were selected from ZINC15 Database (MW ≤ 350 Da and a Log*p* value of −1). Afterwards, a filtering strategy was applied considering different parameters, such as Lipinski “Rule of Five” compliance, risk of potential toxicity (Mutagenic, Tumorigenic, Reproductive, and Irritant effects), topological surface area (TPSA), number of rotatable bonds, and finally, a clustering by structure similarity, providing as a result a total of 52 molecules (Figure 1, Table S1). 

### 2.2. Virtual Screening

Currently, virtual screening has become essential for the drug design process, it permits an accurate prediction of the position and conformation of a ligand in the binding site of a target protein through an established scoring function [20]. After filtering, 52 compounds were docked into the SaSK active site using the protocol described in materials and methods; the five molecules with the highest docking score and structural diversity were selected in this study (Figure 1 and Table 1). 

**Figure 2:**
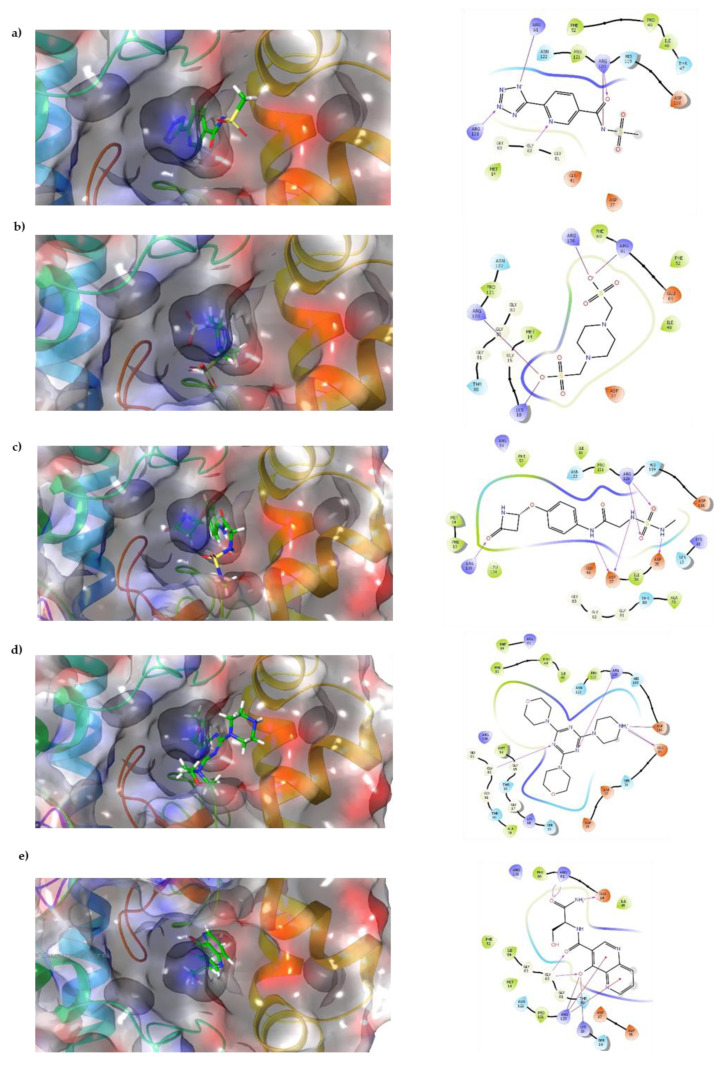
Interaction of top compounds. Two-dimensional representation of SaSK residues interacting with (**a**) C1, (**b**) C2, (**c**) C3, (**d**) C4, and (**e**) C5. Hydrogen bonds are depicted in pink, salt bridges are shown in red-blue, and Pi-cation interactions in red.

Docking results show that compounds interact with residues important for substrate binding or enzyme catalysis, such as Gly82 and Arg138, both are in charge of the stabilization and orientation of shikimate; moreover, Gly83 also participates in substrate stabilization [21]. Conversely, Arg120 is a residue present in the LID domain known for its function as a substrate stabilizing residue in the ATP-shikimate complex [22]. Finally, Asp37 participates in the interaction with the hydroxyl groups of shikimate molecule [23] while Asp35 is an important residue in the nucleotide binding domain that forms hydrogen bonds with other residues that facilitate the approximation of shikimate and ATP [21] (Table 1 and Figure 2).

Although, there are no reports in literature of inhibitors for SaSK, other shikimate kinases, in particular for *M. tuberculosis* and *H. pylori*, have been shown [22,24]. These molecules comprise several scaffolds, such as pyrazolone derivatives, manzamines, 2-aminobenzothiazoles, and substrate analogues, that particularly target the shikimate binding site [25,26,27].

Docking results in this study were able to select compounds that possess a particular orientation that allows them to perform important interactions with residues that are vital for enzyme catalysis, such as Arg61, Arg138, and Gly82, which are reported to be critical for ligand stabilization and orientation in studies performed in *M. tuberculosis* shikimate kinase [21,22,23,28].

### 2.3. Molecular Dynamics Studies

**Figure 3:**
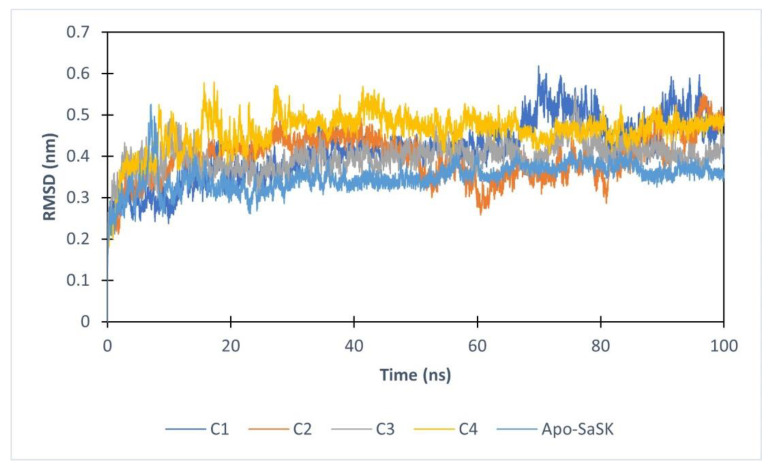
Root mean square deviation analysis for free enzyme and the different protein–ligand complexes.

For purposes of gathering added information about SaSK-potential inhibitor complex, molecular dynamics simulations of 100 ns were performed. First, protein–ligand complex stability was evaluated analyzing the root mean square deviation (RMSD) of the Cα protein atoms. The data show that after 20 ns, four of the complexes reach stability, however, **C5** leaves the binding site after 6 ns of simulation, therefore, no analysis was performed for this compound. Average RMSD values obtained are 0.416, 0.397, 0.397, and 0.459 nm for SaSK-**C1**, SaSK-**C2**, SaSK-**C3**, and SaSK-**C4** complexes, respectively, indicating that all remain stable during simulation time (Figure 3).

**Figure 4:**
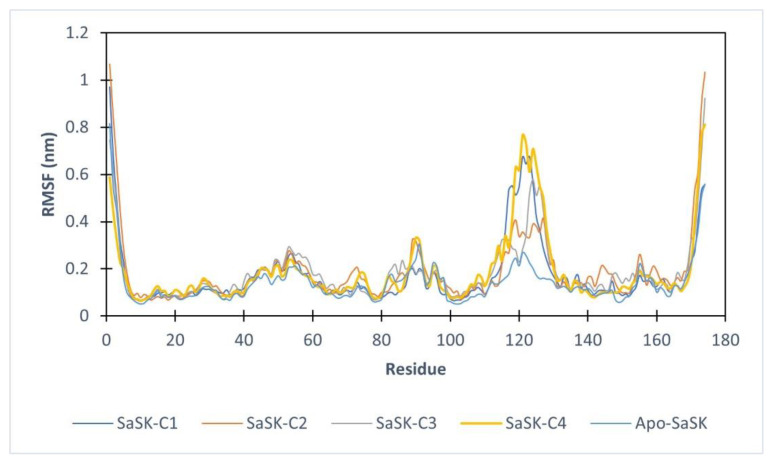
Root mean square fluctuation analysis for free enzyme and the different protein–ligand complexes.

Furthermore, root mean square fluctuations analysis (RMSF) was performed, the results show that in all four complexes, the highest fluctuation observed corresponds to the LID domain (residues 120–135 in SaSK), a region that it is known for its great flexibility [29], in this case, this movement can be attributed to an essential movement to accommodate the compound within the binding site. These fluctuations are more notorious in SaSK-**C1**, SaSK-**C3,** and SaSK-**C4** complexes (Figure 4). Average RMSF values obtained are 0.172, 0.191, 0.184, and 0.186 nm for SaSK-**C1**, SaSK-**C2**, SaSK-**C3**, and SaSK-**C4**, respectively.

**Figure 5:**
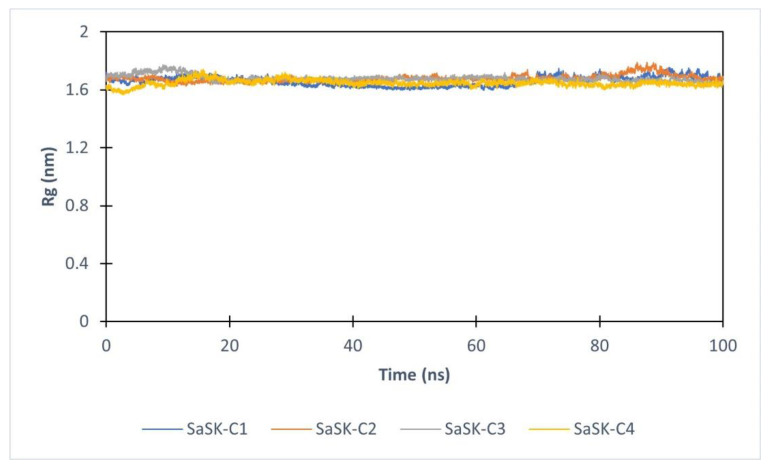
Radius of gyration for SaSK in complex with C1(Blue), C2 (Orange), C3 (Gray), C4 (Yellow).

Furthermore, to assess the effect of compound binding in protein tertiary structure, the radius of gyration (Rg) analysis was realized. As it can be seen, the value of Rg in each complex keeps constant during entire simulation time, suggesting that none of the potential inhibitors alters the structure of the protein (Figure 5).

**Figure 6:**
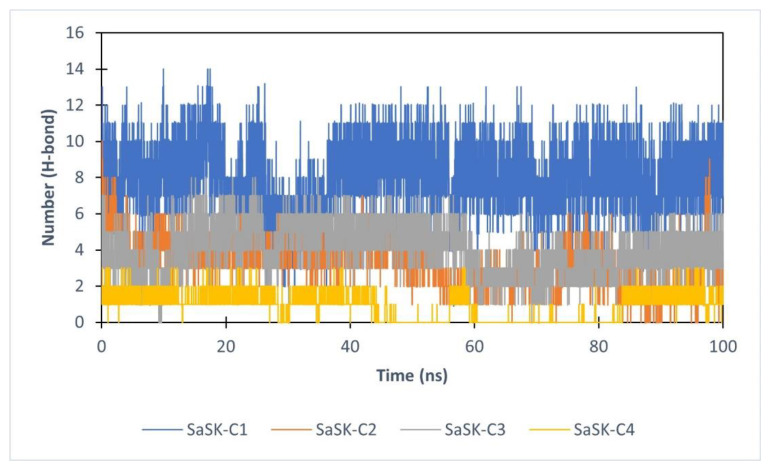
H-Bond analysis for each SaSK–ligand complex.

Finally, hydrogen bond analysis was performed. The data show that the number of H-bonds in the complexes vary during simulation time with an average of 7, 4, 3, and 1 for SaSK-**C1**, SaSK-**C2**, SaSK-**C3**, and SaSK-**C4**, respectively, suggesting a better binding mode for compound **C1** (Figure 6 and Table 2). 

### 2.4. Linear Interaction Energy

The linear interaction energy (LIE) [30] approach is an extensively described method to compute binding affinities. It permits to combine explicit conformational sampling (of the protein-bound and unbound-ligand states) with efficiency to be able to calculate quantitative values for the protein–ligand binding free energy ΔG_bind_.

In this study, the LIE method was employed to calculate the binding affinities of the four complexes evaluated by molecular dynamics. Results show that **C1** and **C4** obtained a negative value for ΔG_bind_ because of a combination of Van der Waals and electrostatic interaction energies, while **C2** and **C3** obtained a positive one, indicating that the former make a more stable complex with SaSK than the latter (Table 3). 

### 2.5. ADME-Tox Evaluation

In the final analysis to complete the characterization of these potential inhibitors, an important issue during first steps of drug design process is the prediction of the ADME-Tox properties of the molecules. In this context, a detailed study was performed for the four compounds using the SwissADME online tool [31] and PreADMET server [32]. The data show that, in general, all compounds obtained evaluations in the permitted range of each characteristic, which indicates the drugability potential of these compounds (Table 4 and Table 5). It is important to note that this type of characterization has not been reported for inhibitors against other SKs from bacteria [22,24].

## 3. Materials and Methods

### 3.1. Small Molecules Chemical Library

In this study, A 3D small molecule database was retrieved from ZINC15 database Tranches (http://zinc15.docking.org/, accessed on 9 January 2021) [33]. First, based on substrate structure, a Log *p* value of −1 and a molecular weight ≤ 350 Da, were used as selection criteria. By the time this work was finished, the number of compounds was neighboring 5 million.

Prior to virtual screening by docking, compounds were filtered according to Lipinski’s Rule of Five’ [34] to select those that possess physicochemical properties present in potential drug candidates only. Additionally, given that unfavorable structural alerts that can produce toxicity may lead to a compound being rejected in further studies [19,35], an in silico toxicity risk assessment for Mutagenicity, Tumorigenic, Irritant, and Reproductive effects was performed using Osiris Data Warrior software [36]. The presence of a single toxic parameter was enough to eliminate a given compound. Furthermore, the topological surface area (TPSA) was also calculated where a value ranging from >75 to <140 was necessary to be included, along with a number of rotatable bonds of less than 5. Finally, compounds were clustered according to their structure and similarity using as criteria that the highest similarity value fell below 0.8 according to Data Warrior Software.

### 3.2. Docking Studies

The previously reported SaSK 3D homology model [18] was employed for docking studies, the protein structure was prepared with the Protein Preparation Wizard in Maestro (Schrödinger Suite Release 2019-4) [37]. Bonds order was assigned, hydrogen atoms were added, and formal charges were treated, protein minimization was applied with the OPLS3e forcefield. The grid box was generated with default settings, with a 10Å × 10Å × 10Å size, using the center between amino acids Met14, Asp37, Ile48, Phe60, Arg61, Gly81, Gly82, Gly83 Pro118, and Arg138, which correspond to amino acids forming the shikimate binding site of the enzyme [26,29]. After filtering criteria (Figure 1), the 3D ligand structures of compounds selected were prepared using Ligprep, at a selected pH range of 7 ± 2, where ionization states were generated; the energy was minimized using the OPLS3e force field. Docking studies were carried out with Glide [38] implemented in the Maestro software, using the extra precision (XP) mode [39] that provides further elimination of false positives by applying extensive sampling and a more stringent scoring function, the best five poses of each compound were retained as output.

### 3.3. Molecular Dynamics Studies

Molecular dynamics simulations were performed using GROMACS version 2019.3 [40] and CHARMM 36 forcefield [41]. Before MD simulation, compounds were parametrized using SwissParam Server (http://swissparam.ch/, accessed 17 May 2021) [42]. SaSK coordinates and topology were constructed using GROMACS, then each ligand was merged into a complex with SaSK and the system was immersed into the center of a dodecahedral box, the solute box distance was set at 1.0 nm. The system was solvated by the addition of TIP3P waters [43] and counterions were added to reach a salt concentration of 0.15 M. 

MD simulations began with an energy minimization (EM) simulation as the first step, which was performed during 100 ps to reach a local minimum employing the steepest descent algorithm. Afterwards, the system was submitted to temperature and pressure equilibration by performing two 100 ps equilibration steps namely, an isothermal-isochoric (NVT) ensemble followed by an isothermal-isobaric (NPT) ensemble under no position restraint, thus bringing the system to a 310 K temperature and 1 bar pressure. Temperature and pressure were maintained by employing the velocity-rescale thermostat [44] and the Parrinello-Rahman pressure coupling methods [45]. Finally, a 100 ns timescale MD was carried out, employing a 1.2 nm for short-range interactions and the leap-frog integrator algorithm. MD simulations were then analyzed using Visual Molecular Dynamics (VMD) software [46]. Furthermore, to explore the structural and dynamic behavior of the protein-ligand complex, analysis of the MD data involved root mean square deviation (RMSD), root mean square fluctuation (RMSF), radius of gyration (Rg), and hydrogen bond analysis (H-bonding). 

### 3.4. Linear Interaction Energy Calculations

Binding free energies were obtained for each of the complexes based on the linear interaction energy (LIE) method calculated by the Equation (1):

∆G_bind_ = α[(V_LJ_)_bound_ − (V_LJ_)_free_] + β[(V_CL_)_bound_ − (V_CL_)_free_] + γ (1)

where (V_LJ_)_bound_ indicates the average Lennard–Jones energy for ligand–protein interaction; (V_LJ_)_free_ is the average Lennard–Jones energy for ligand–solvent interaction; (V_CL_)_bound_ is the average electrostatic energy for ligand–protein interaction; (V_CL_)_free_ is the average electrostatic energy for ligand–solvent interaction; the LIE coefficients are given by α, β, and γ which for small drug-like ligands correspond to α = 0.18, β = 0.50, and γ = 0.00 [30,47,48].

### 3.5. ADME Properties Prediction

Absorption, distribution, metabolism, and excretion properties of each potential inhibitor were predicted using SwissADME web tool [31] and the online PreADMET server (http://preadmet.bmdrc.org, accessed 23 August 2021) [32].

## 4. Conclusions

The study of an around 5 million small molecules database through a computer-assisted drug design strategy, permitted to find the first set of potential inhibitors of SaSK. According to the structural analysis, these compounds formed interactions with residues important for enzyme catalysis. Furthermore, they demonstrated good ADME-Tox and druglike characteristics, which make these molecules an attractive starting point for the development of new drugs against MRSA.

## Figures and Tables

**Table 1:** Glide XP docking results for the top five hits.

Compound	Zinc ID	Structure	Docking Score (kcal/mol)	Interacting Residues (Distance Cut-Off 4.0Å)
**C1**	000737165696	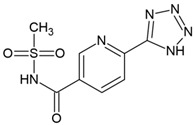	−3.905	Arg61 ^2^, Gly82 ^1^, Arg120 ^1, 2^, Arg138 ^1^, Met14 ^4^, Asp37 ^4^, Glu41 ^4^, Thr47 ^4^, Ile48 ^4^, Phe49 ^4^, Phe52 ^4^, Gly81 ^4^, Gly83 ^4^, Asp116 ^4^, His119 ^4^, Pro121 ^4^, Asn122 ^4^
**C2**	000019366016	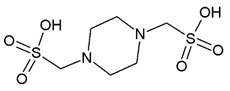	−3.846	Lys18 ^2^, Arg61 ^2^, Arg120 ^2^, Arg138 ^2^, Met14 ^4^, Gly15 ^4^, Lys18 ^4^, Asp37 ^4^, Ile48 ^4^, Phe52 ^4^, Phe60 ^4^, Glu64 ^4^, Thr80 ^4^, Gly81 ^4^, Gly82 ^4^, Gly83 ^4^, Pro121 ^4^, Asn122 ^4^
**C3**	000653035164 S enantiomer	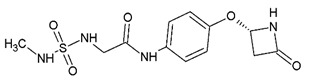	−3.451	Asp35 ^1^, Asp37 ^1^, Arg120 ^1^, Arg138 ^1^, Phe13 ^4^, Met14 ^4^, Lys18 ^4^, Ser19 ^4^, Ile36 ^4^, Glu41 ^4^, Ile48 ^4^, Phe52 ^4^, Arg61 ^4^, Ala79 ^4^, Thr80 ^4^, Gly81 ^4^, Gly82 ^4^, Gly83 ^4^, Asp116 ^4^, His119 ^4^, Pro121 ^4^, Asn122 ^4^
**C4**	000000197090	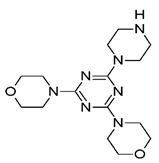	−3.406	Glu41 ^1,2^, Gly82 ^1^, Asp116 ^2^, Arg120 ^1^, Met14 ^4^, Gly15 ^4^, Thr16 ^4^, Gly17 ^4^, Lys18 ^4^, Ser19 ^4^, Asp35 ^4^, Asp37 ^4^, Ser38 ^4^, Ile48 ^4^, Pro49 ^4^, Phe52 ^4^, Phe60 ^4^, Arg61 ^4^, Ala79 ^4^, Thr80 ^4^, Gly81 ^4^, Gly83 ^4^, His119 ^4^, Pro121 ^4^, Asn122 ^4^, Arg138 ^4^
**C5**	001153862505 R enantiomer	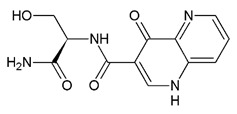	−3.286	Lys18 ^2^, Arg61 ^1^, Glu64 ^1^, Gly82 ^1^, Arg120 ^3^, Met14 ^4^, Ser19 ^4^, Asp35 ^4^, Asp37 ^4^, Ile48 ^4^, Phe52 ^4^, Phe60 ^4^, Thr80 ^4^, Gly81 ^4^, Gly83 ^4^, Ile84 ^4^, Pro121 ^4^, Asn122 ^4^, Arg138 ^4^

^1^ H-bond interaction; ^2^ Salt Bridge type interaction; ^3^ Pi-cation type interaction; ^4^ Hydrophobic interactions^.^

**Table 2:** Interacting residues through the 100 ns simulation time at selected time frames.

	0 ns	20 ns	40 ns	60 ns	80 ns	100 ns
**C1**	* Arg61, Gly82, Arg138	* Gly15, Lys18	* Gly15, Lys18, Arg61	* Gly15, Lys18, Arg138	* Gly15, Lys18, Arg138	* Gly15, Lys18, Arg138
**C2**	* Lys18, Arg61, Gly82, Arg120 Asn122, Arg138	* Lys18, Arg61, Gly82	* Lys18, Arg61, Gly82, Asn115	* Lys18, Gly82, Lys126	* Asn122, Lys 126, Arg138	* Thr127
**C3**	* Lys18, Asp37, Arg120, Arg138	* Ser19, Asp35, Gly82, Arg120, Asn124	* Ser19, Asp35, Arg120, Asn124	* Asp35, Arg120, Ala123	* Ser19, Asp35, Arg61	* Ser19, Asp35, Arg120, Asn122
**C4**	*Ser19, Glu41, Arg120	*Glu41, Gly82	*Glu41, Arg120	*Asp37, Glu41	*Glu41, Gly82	*Ser19, Glu41 ,

* H-bond interactions.

**Table 3:** Binding free energies calculated by the LIE method for each complex in MD simulation.

	Energy (Kcal/mol)				
Complex	(V_LJ_)_Bound_	(V_LJ_)_Free_	(V_CL_)_Bound_	(V_CL_)_Free_	ΔG_bind_ kcal/mol
SaSK-C1	−21.39	−5.46	−107.92	−70.41	−21.62
SaSK-C2	−14.45	1.96	−65.15	−160.90	48.41
SaSK-C3	−13.93	−4.69	−6.74	−8.58	0.8
SaSK-C4	−23.25	−14.70	−28.31	−43.56	−37.47

(V_LJ_)bound: average Lennard–Jones energy for ligand–protein interaction; (V_LJ_)free: average Lennard-Jones energy for ligand-solvent interaction; (V_CL_): average electrostatic energy for ligand-protein interaction; (V_CL_)free: average electrostatic energy for ligand–solvent interaction.

**Table 4:** Physicochemical properties of potential SaSK inhibitors.

	C1	C2	C3	C4
MW (g/mol)	266.24	272.30	328.34	335.4
RB	4	4	5	3
HBA	9	8	7	6
HBD	0	0	4	1
MR	58.9	59.83	81.04	102.64
TPSA (Å^2^)	124.04	137.64	134.01	78.88
cLogP	0.47	−2.60	0.68	2.96
Lipinski rules violations	0	0	0	0
Water Solubility				
LogS	−1.06	2.84	−1.0	−1.87
Class	Very soluble	Highly soluble	Very soluble	Very soluble
Druglikeness				
Ghose	Yes	1 violation: WLOGP < −0.4	1 violation: WLOGP<-0.4	No; 1 violation: WLOGP < −0.4
Veber	Yes	Yes	Yes	Yes
Egan	Yes	1 violation: TPSA > 131.6	1 violation: TPSA > 131.6	Yes
Muegge	Yes	1 violation: XLOGP3 < −2	Yes	Yes
Bioavailability Score	0.56	0.55	0.55	0.55
Medicinal Chemistry				
PAINS	No alerts	No alerts	No alerts	No alerts
Brenk	No alerts	1 alert: sulfonic_acid_2	No alerts	No alerts
Leadlikeness	Yes	Yes	No	Yes
Synthetic accessibility	2.21	3.12	3.19	3.16

* All values were calculated with SwissADME web tool. Molecular weight (MW: 50–500 Da), number of rotatable bonds (RB: 0–5), number of hydrogen acceptors (HBA: 0–10), number of hydrogen donors (HBD: 0–5), Molar refractivity (MR: 40–130), Topological Polar Surface Area (TPSA: 20–130), octanol/water partition coefficient (cLOGP: −2 to 10), Lipinski, Ghose, Veber, Egan, and Muegge (Filters that determine druglikeness of a compound: no violations are considered ideal), Number of Brenk alert and PAINS alert ( number of alerts for undesirable substructures/substructures, a result with No alerts is ideal), Synthetic accessibility (Ease of compound synthesis: score ranges from 1 that indicates very easy to 10 very difficult).

**Table 5:** PreADMET results characteristics for potential SaSK inhibitors.

	C1	C2	C3	C4
BBBP	0.0488584	0.0466103	0.037606	0.0466103
CaCo-2 (nm/s)	4.65788	2.24237	0.373322	2.24237
HIA (%)	64.622234	58.373794	69.411618	58.373794
MDCK (nm/s)	1.05816	354.049	0.591682	354.049
In vitro P-glycoprotein inhibition	Non	Non	Non	Non
PPB (%)	65.392204	45.848496	39.567509	45.848496
Water solubility in pure water (mg/L)	9254.72	2.96157e + 006	3141.21	2.96157e + 006
In vitro skin permeability (logKp, cm/h)	−2.71782	−2.46455	−4.61446	−2.46455

* All values were calculated with PreADMET server. BBBP, in vivo Blood-Brain Barrier Penetration (less than 0.1, low absorption to Central Nervous System; 0.1–2, medium absorption), CaCo-2, in vitro CaCo-2 cell permeability (4–70 nm/s, middle permeability; more than 70 high permeability); HIA, Human Intestinal Absorption (20–70% moderately absorbed compounds; 70–100% well absorbed compounds); MDCK, in vitro MDCK cell permeability (4–70 nm/s, middle permeability); In vitro P-glycoprotein inhibition (substrate or non-substrate of the permeability glycoprotein, a negative result is ideal), PPB, in vivo Plasma Protein Binding (less than 90%, chemicals weakly bound); In vitro skin permeability (logKp, cm/hour, the more negative the log *K_p_* the less skin permeant is the molecule).

## Data Availability

Not applicable.

## Supplementary Materials

The following is available online. Table S1: Structures of the compounds selected for virtual screening.
