# Supplementary material for: Finding the First Potential Inhibitors of Shikimate Kinase from Methicillin Resistant Staphylococcus aureus through Computer-Assisted Drug Design
Source: Molecules. 2021 Nov 8;26(21):6736. doi: 10.3390/molecules26216736 (PMC8587801; doi:10.3390/molecules26216736)
Supplement: Supplementary file 1 [file molecules-26-06736-s001.zip › molecules-1456799-supplementary.pdf]

Table S1. Structures of the compounds selected for virtual screening.

ZINC ID	Name	Structure
ZINC000000033762	4-(1,2-dimethyl-3,5-dioxo-1,2,4-triazolidin-4-yl)benzenesulfonamide	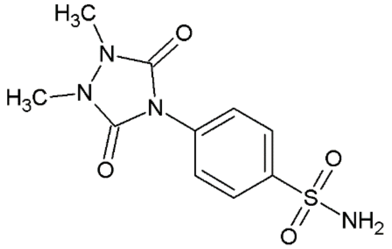
ZINC000001663005	(1*R*,2*S*,3*R*)-1-(1,2,4-benzotriazin-3-yl)butane-1,2,3,4-tetrol	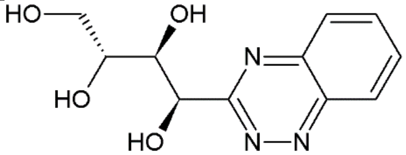
ZINC000019594727	1,4,7,10,13,16-hexazacyclooctadecane	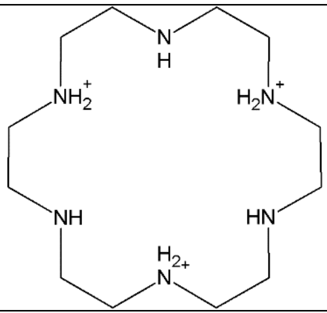
ZINC000039404119	methyl 3-[bis(methylsulfonyl)amino]propanoate	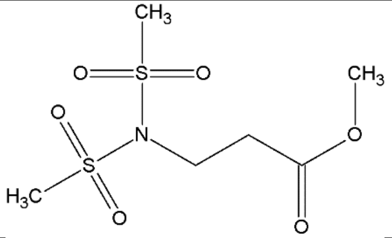
ZINC000090475824	1,2,4,5-Tetrazine, 3,6-bis(ethylsulfonyl)-	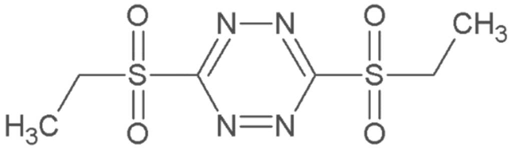
ZINC000863703032	*N*-{(2*R*)-1-[methoxy(methyl)amino]-1-oxopropan-2-yl}-1,3-dimethyl-2,4-dioxo-1,2,3,4-tetrahydropyrido[2,3-*d*]pyrimidine-7-carboxamide	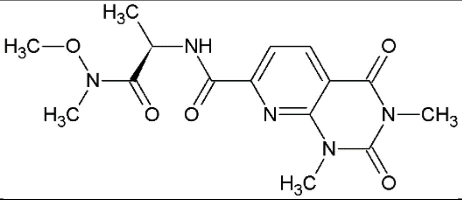
ZINC001156694532	(4*R*)-4-{[3-(piperazin-1-ylcarbonyl)pyridin-2-yl]amino}isoxazolidin-3-one	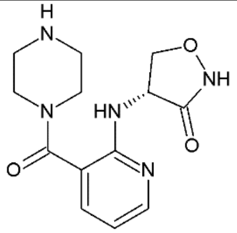

ZINC000938450483	*N*-[(3*S*)-1-{[(4*S*)-2-oxoimidazolidin-4-yl]carbonyl}pyrrolidin-3-yl]-1*H*-1,2,4-triazole-3-carboxamide	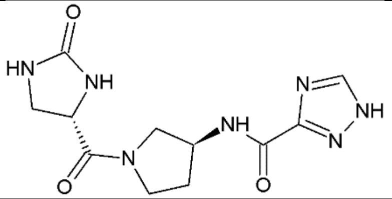
ZINC000362648055	(4*S*,4'*S*)-4,4'-{[6-(methylimino)-1,3,5-triazinane-2,4-diylidene]dinitrilo}dipyrrolidin-2-one	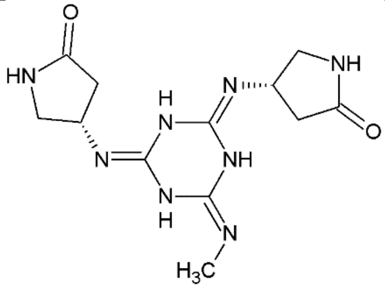
ZINC000852816657	*N*-[2-(benzylamino)-2-oxoethyl]-3-hydroxyazetidine-3-carboxamide	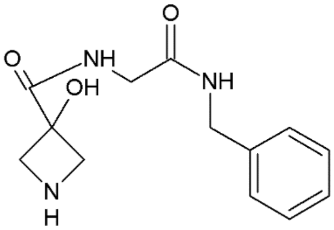
ZINC000624224393	(2*R*)-3-[(4,6-diimino-1,3,5-triazinan-2-ylidene)amino]-2-(pyridin-3-yl)propan-1-ol	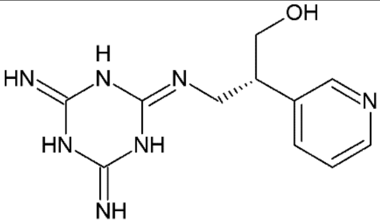
ZINC000941661806	3-[(4-{1-[(2*S*)-2-hydroxypropyl]azetidin-3-yl}piperazin-1-yl)carbonyl]pyrazine-2-carboxamide	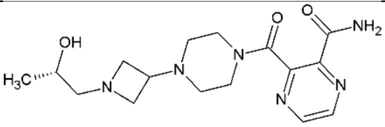
ZINC000493041505	(2*Z*)-3-(1,3-dimethyl-2,4-dioxo-1,2,3,4-tetrahydropyrimidin-5-yl)-*N*-[2-(dimethylsulfamoyl)ethyl]prop-2-enamide	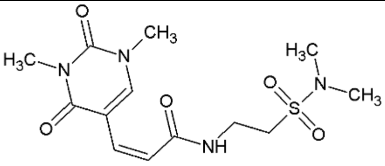
ZINC000576768709	(3*R*,4*R*)-4-methoxy-1-{[6-(methylsulfonyl)pyridin-3-yl]sulfonyl}pyrrolidin-3-ol	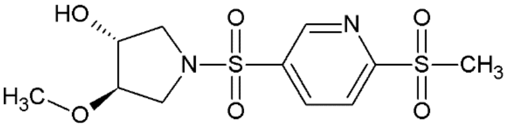
ZINC000262531716	N-[2-(Methylamino)ethyl]-4-(3-oxopiperazin-1-yl)sulfonylbenzamide	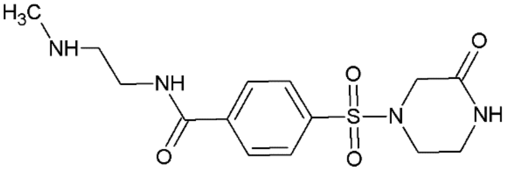

ZINC001158970356	(2*R*,3*R*,4*R*,5*S*,6*R*)-6-(hydroxymethyl)-3-[(4-methoxypyridin-2-yl)amino]tetrahydro-2*H*-pyran-2,4,5-triol	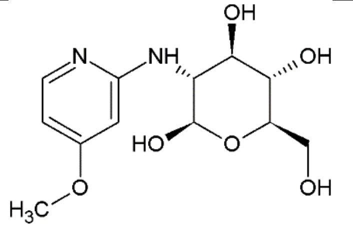
ZINC000353827182	*N*-methyl-*N'*-[(3*E*)-2-methyl-1,2-dihydro-3*H*-1,2,4-triazol-3-ylidene]benzene-1,3-disulfonamide	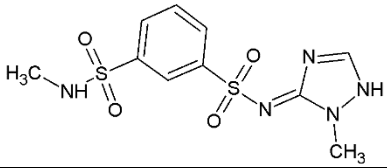
ZINC000583151023	*N*-{2-[(dimethylsulfamoyl)amino]ethyl}-1,3-dioxo-2,3-dihydro-1*H*-isoindole-5-carboxamide	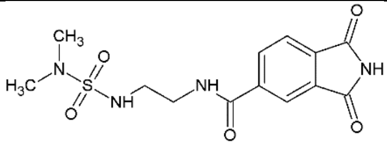
ZINC000623936890	*N*-(4,6-diimino-1,3,5-triazinan-2-ylidene)-1-[2-(morpholin-4-yl)ethyl]-1*H*-pyrazol-4-amine	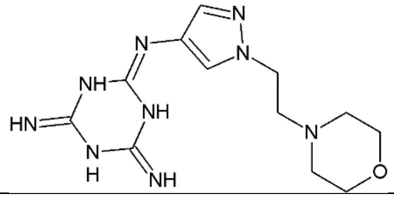
ZINC001116751280	2-{[(6-bromo-5-methoxypyridin-3-yl)carbonyl]amino}propanediamide	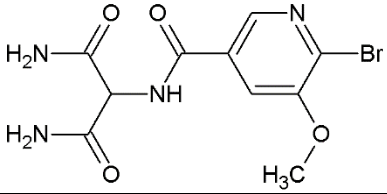
ZINC001259045039	*N*-(2-amino-2-oxoethyl)-2-{[(2,4,5-trifluorophenyl)sulfonyl]amino}acetamide	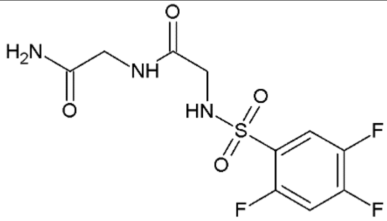
ZINC000517234699	2-amino-*N*-(2-{[2-(dimethylamino)pyrimidin-5-yl]amino}-2-oxoethyl)acetamide	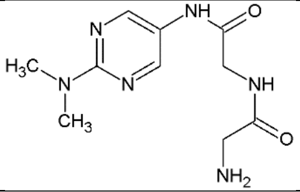
ZINC001219697045	(3a*R*,9b*S*)-*N*-[2-(3-hydroxyazetidin-3-yl)ethyl]-5-oxo-2,3,3a,4,5,9b-hexahydro-1*H*-pyrrolo[3,4-*c*]isoquinoline-8-carboxamide	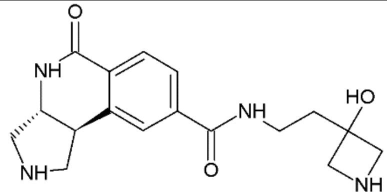

ZINC001364199721	4-[3-(aminomethyl)pyrazin-2-yl]-*N*-methylpiperazine-1-sulfonamide	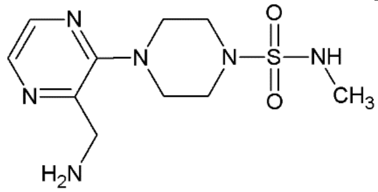
ZINC000012322217	2,5,8-Trimethyl-1H-dipyrrolo[3,4-e:3',4'-g]isoindole-1,3,4,6,7,9(2H,5H,8H)-hexone	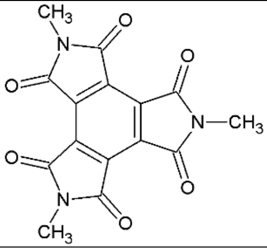
ZINC001329482875	2-[4-(2-hydroxyethyl)piperazin-1-yl]-*N*-methylethanesulfonamide	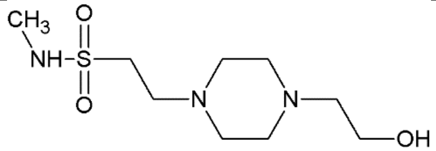
ZINC001346492570	(3*S*)-3-hydroxy-1-[(5-hydroxypyridin-3-yl)acetyl]pyrrolidine-3-carboxamide	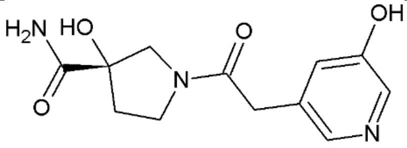
ZINC000517602023	(2*R*)-3,3,3-trifluoro-*N*,*N*-dimethyl-2-{[(5*E*)-1-methyl-1,2-dihydro-5*H*-tetrazol-5-ylidene]amino}propane-1-sulfonamide	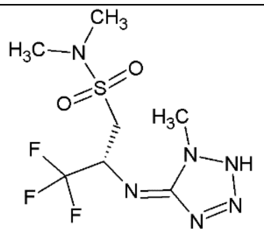
ZINC001423678455	*N*-[dimethyl(oxido)-l^{6}-sulfanylidene]-*N*'-[2-(ethylsulfonyl)ethyl]sulfuric diamide	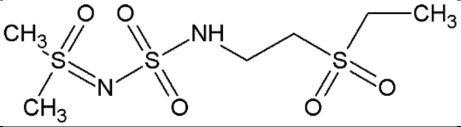
ZINC000857210028	6-bromo-3-[2-(4,5-dihydro-1*H*-imidazol-2-yl)hydrazinyl]-5-methoxy-2*H*-indol-2-one	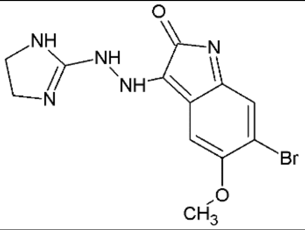
ZINC001168918857	1-{1-[4-(methylsulfonyl)pyridin-2-yl]-1*H*-tetrazol-5-yl}methanamine	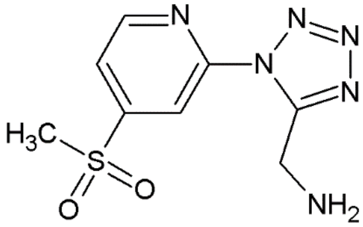

ZINC001167923698	2-[(2-chloro-6,7-dihydro-5*H*-pyrrolo[3,4-*d*]pyrimidin-4-yl)amino]-2-(hydroxymethyl)propane-1,3-diol	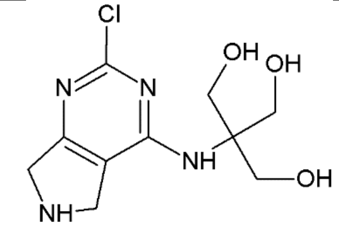
ZINC000972896897	*N*-{*trans*-3-[(pyridin-4-ylcarbonyl)amino]cyclobutyl}ethanediamide	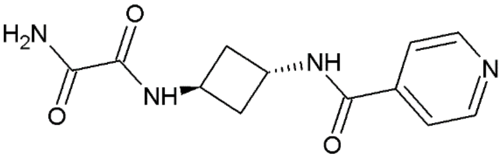
ZINC001364023987	*N*'-{[(3*R*,4*R*)-4-hydroxytetrahydrofuran-3-yl]sulfamoyl}-*N*,*N*-dimethylmethanesulfonimidoamide	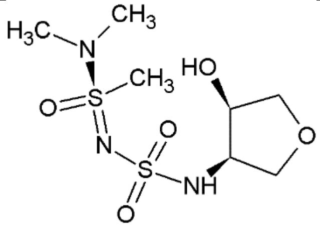
ZINC000808047782	(2*S*)-3-{[2-amino-6-(1*H*-pyrazol-1-yl)pyrimidin-4-yl]amino}-2-hydroxy-*N*-methylpropanamide	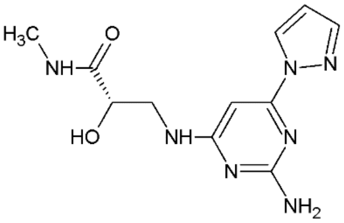
ZINC001363986270	3-[{[(dimethylamino)(methyl)oxido-l^{6}-sulfanylidene]sulfamoyl}(methyl)amino]propanamide	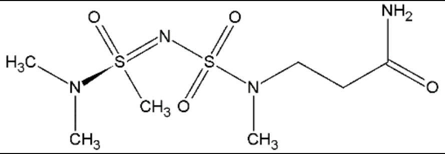
ZINC000547286627	(4*E*)-4-{[(2*S*)-2-sulfamoylpropyl]imino}-1,4-dihydropyridine-3-carboxamide	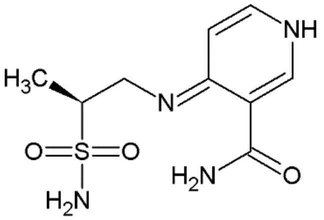
ZINC001158867950	6-(6-methyl-2,6-diazaspiro[3.3]hept-2-yl)pyridazine-3-sulfonamide	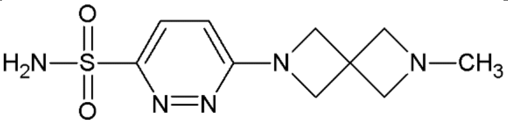
ZINC000425289994	(1*R*,2*R*,1'*R*,2'*R*)-2,2'-{[6-(methylimino)-1,3,5-triazinane-2,4-diylidene]dinitrilo}dicyclobutanol	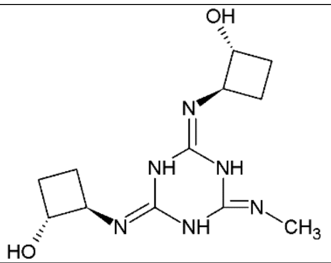

ZINC000624085464	4-{[(4,6-diimino-1,3,5-triazinan-2-ylidene)amino]methyl}-*N*,*N*-dimethylpyrimidin-2-amine	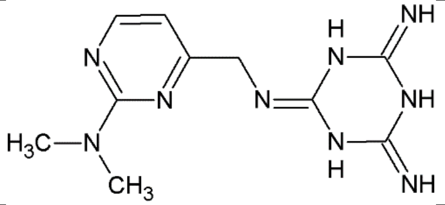
ZINC001364169073	(3*R*)-3-{[(4*Z*)-1,2-dihydro-4*H*-pyrazolo[3,4-*d*]pyrimidin-4-ylideneamino]methyl}tetrahydrothiophene-3-ol 1,1-dioxide	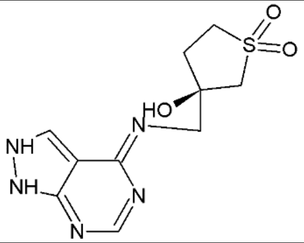
ZINC001166935835	1-[2-(methylsulfonyl)-6,7,8,9-tetrahydro-5*H*-pyrimido[4,5-*d*]azepin-4-yl]azetidin-3-ol	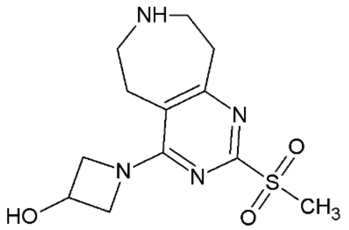
ZINC001118094695	(2*Z*)-*N*-[(2-amino-2-oxoethyl)sulfonyl]-3-[(2*R*)-tetrahydrofuran-2-yl]prop-2-enamide	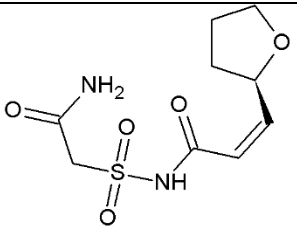
ZINC000648129059	*N*-[(2*R*)-2-aminopropyl]-2-sulfamoylpyridine-4-carboxamide	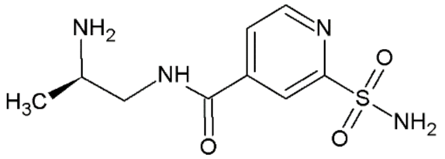
ZINC000005582647	4,7-dihydro-4,7-phenanthroline-1,2,3,8,9,10-hexone	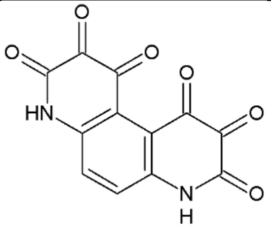
ZINC000002865127	1,5-dihydroxy-4,4,8,8-tetramethoxy-4*H*,8*H*-[1,2,5]oxadiazolo[3,4-*f*][2,1,3]benzoxadiazole-1,5-diium	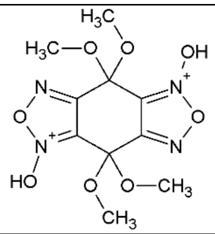
